# O10 Multi-disciplinary working in SpA diagnosis: case study

**DOI:** 10.1093/rap/rkab067.009

**Published:** 2021-10-19

**Authors:** Fraser Morgan, Matthew Virden, John Etherington

**Affiliations:** 1 St George's University, London, United Kingdom; 2 Pure Sports Medicine, London, United Kingdom

## Abstract

**Case report - Introduction:**

High-intensity exercise is effective in treating Axial Spondyloarthritis (SpA). A reduction in morbidity therefore should be gained from accurate diagnosis early in the disease history.

In 2021 the National Axial Spondylarthritis Society (NASS) released a statement to reduce time to diagnosis (TTD) of Axial SpA from 8 years to 1, to reduce morbidity and long-term complications of chronic inflammation.

This case study aims to provide an example of how a multidisciplinary team working in close clinical proximity (CCP MDT) can be utilised to reduce the TTD and optimise treatment and biomechanics of acute and chronic SpA.

**Case report - Case description:**

Cases 1 and 2 compare patients presenting to a musculoskeletal clinic with lower back pain, stiffness, and reduced range of movement.

Case 1. Subacute presentation: 25-year-old male with a 3-month history of lower back pain and stiffness. This progressed to a month of not being able to walk, disturbed sleep and morning stiffness of > 1hr. Clinical examination showed no extra musculoskeletal manifestations, and no other joint pain. Tests showed HLA B27 positive status with florid bilateral sacroiliitis on MRI.

Case 2. Chronic presentation: 34-year-old male with 20-year history of insidious onset lower back pain previously diagnosed as “non-specific” back pain by a series of health care professionals (HCPs). HLA B27 negative, MRI showed acute on chronic SIJ arthropathy with juxtarticular sclerosis.

Patients were seen by a HCP who referred to a rheumatologist. In both, clinical and radiological signs indicated Axial SpA which was treated with 120mg of IM Depo-Medorone and 8-weeks of strength-based active intervention from a physiotherapist and strength and conditioning coach. BASDAI, 5-rep max single leg press scores were recorded pre- and post-intervention (Table 1.).

**Case report - Discussion:**

Conventional therapies for SpA back pain are effective even in chronic cases; this is reflected in the improvements made across both cases. Using a similar approach of high-intensity exercise and mobility both were able to significantly improve BASDAI scores and lower limb strength.

We can hypothesise that attempts to innovate the biomechanics of the treatments is missing the fundamental issue surrounding SpA back pain highlighted by NASS; TTD takes too long for too many.

There is a feasible argument therefore that it is the structures of how we operate as HCPs that is reducing the efficacy of said treatments. This is evidenced by case 2 (Table 1), whose multiple presentations to different HCPs over 20 years led to potentially preventable prolonged morbidity as well as increased the risk of future complications due to chronic inflammation. This is a story that is sadly reflective of the disease histories of many SpA patients.

Much like any primary care provider, patients at this clinic will first present to a HCP. The difference is that all HCPs are trained to recognise features of SpA back pain and encouraged to flag concerns to a doctor.

HCPs work in adjacent spaces allowing for better sharing of ideas and concerns that is often reduced by physical separation. This creates the concept of a CCP MDT. HCPs are encouraged to share concerns face to face without reproach or the formality or delay of a written request. This structure has proven to be effective in diagnosis in both of these cases but has proven to be as useful in many more instances to rule out a differential or allay a concern.

These cases argue that the low lying fruit of improving clinical outcomes in this cohort is addressing how we as HCPs interact and cooperate.

**Case report - Key learning points:**

Biomechanics of the treatments of SpA are effective in the majority of cases when applied by sufficiently skilled practitioners. It is the TTD that we should address to aid the reduction in morbidity in this patient group through the following recommendations:

Foster education of SpA in allied health professionals. This allows us to be aware of the boundaries of our own knowledge and at what point a different clinical perspective should be sought.

Diversify clinical environments and encourage MDT working. The use of CCP MDT structures allows more nuanced access to different opinions in a safe supportive manner, allowing for sharing of ideas and concerns – allowing for supporting diverse expertise and insight.

If we are to reduce TTD to the 1-year NASS hopes for, we should first look at how we work, not what we do.

Ongoing research and auditing of patients is being carried out to better assess how educational practices can be optimised to help meet the NASS targets. For more information or to collaborate contact matthew.virden@puresportsmed.com

**Table:** 

Case 1	Pre-intervention	Post-intervention
BASDAI	5.1	0.9
Single leg press 5RM	55kg	75kg

**Table:** 

Case 2	Pre-intervention	Post-intervention
BASDAI	6.1	4.2
Single leg press 5RM	65kg	115kg

